# Age at menarche and ischemic heart disease: An update mendelian randomization study

**DOI:** 10.3389/fgene.2022.942861

**Published:** 2022-11-03

**Authors:** Jing Chen, Heng Chen, Qiaozhen Zhu, Qiannan Liu, Yan Zhou, Lan Li, Yan Wang

**Affiliations:** ^1^ The First College of Clinical Medicine, Zhejiang Chinese Medical University, Hangzhou, China; ^2^ Department of Cardiology, The First Affiliated Hospital, College of Medicine, Zhejiang University, Hangzhou, China; ^3^ Clinical Medical School, Henan University, Kaifeng, China; ^4^ Department of Pediatrics, The First Affiliated Hospital of Zhejiang Chinese Medical University, Hangzhou, China

**Keywords:** age at menarche, ischemic heart disease, mendelian randomization, causality, single nucleotide polymorphisms

## Abstract

**Background:** Although earlier menarche age has been associated with ischemic heart disease in previous observational studies, the relationship’s causation has not been shown. Through two-sample Mendelian randomization (MR), we were able to define the causal connection.

**Methods:** We performed Mendelian Randomization (MR) analysis to explore the associations between genetically predicted AAM and IHD. Summary-level databases for exposure and outcome were selected from the MR-Base database (https://gwas.mrcieu.ac.uk/). Single-nucleotide polymorphisms (SNPs) connected to AAM at genome-wide significance level (*p* < 5 × 10^−8^) were considered as instrumental variables (IVs). We used four methods to pool MR estimates, including fixed-effects inverse variance weighting (fe-IVW), multiplicative random-effects inverse variance weighting (mre-IVW), weighted median (WM), and MR-Egger regression methods. Sensitivity analyses were performed to evaluate the robustness of the results. PhenoScanner searches and Multivariable Mendelian randomization (MVMR) analysis was used for assessing confounders.

**Results:** 117 SNPs significantly correlated with AAM were screened as instruments, the results of three main methods showed that genetically earlier AAM may have a causal effect on the higher risk of IHD (fe-IVW: OR = 0.80, 95% CI: 0.72–0.88, *p* < 0.001; mre-IVW: OR = 0.80, 95% CI: 0.70–0.90, *p* < 0.001; WE: OR = 0.79, 95% CI: 0.66–0.93, *p* = 0.006). These results were consistent across sensitivity analyses. MR analysis revealed that there was still a relationship between AAM and IHD even when pleiotropic SNPs of confounders were removed employing PhenoScanner searches. In MVMR, the significant association remained after adjusting for biological sex, but it was attenuated with adjustment of body mass index including childhood and adult.

**Conclusion:** Our MR analysis revealed a substantial genetically determined confounder-mediated relationship between an increase in genetically predicted AAM and a lower risk of IHD. By addressing the intervention of body mass index, the risk of IHD may be lowered.

## Introduction

Menarche has a distinctive function during a girl’s youth as a lifetime marker of first menstruation. Because of this, the age at menarche (AAM) is typically well remembered in adulthood, and many epidemiological studies, therefore, prefer to see it as an essential object of study (Parent et al., 2003). In some reports (Day et al., 2015b; Chan et al., 2022), AAM has been used as a proxy for the time of pubertal maturation or the onset of puberty to explore the impact of earlier or later puberty on cardiovascular health in adults, such as hypertension, angina and heart attacks in later life. It has also been shown in several studies that early AAM does increase the likelihood of cardiovascular illnesses (Lakshman et al., 2009; Ibitoye et al., 2017).

Despite advances in disease prevention and diagnosis, an increasing number of women are still diagnosed with or die from cardiovascular disease each year (Mosca et al., 2011; Zheng et al., 2021). Ischemic heart disease (IHD) is the main cause of mortality in women worldwide, yet because of a lack of understanding of gender differences and inadequate management systems, more women than men pass away from IHD every year (Davies and Rier, 2018; Schmidt et al., 2018; Majidi et al., 2021). As cardiovascular risk factors, several physiological issues unique to women, such as early menopause, polycystic ovary syndrome, eclampsia, and early menarche, are currently increasingly being researched (Ahmed et al., 2014; Mehilli and Presbitero, 2020). These studies also include research on the correlation between AAM and IHD (Schmidt et al., 2018).

Mendelian randomization (MR) was proposed by Katan in 1986 to exploit the causal association between phenotypes and diseases through the use of genotypes as instrumental variables (Katan, 1986). As an analytical method using ready-made epidemiological data to identify causal estimates (Davies et al., 2018), MR carries out the genetic instruments that are fixed before birth, so confounding factors or reverse causality has little influence on instrumental genetic predisposition. Genetic predisposition can contribute to the occurrence of a target exposure, and if a significant association exists between the genetic predisposition and outcome, it suggests a causal effect of exposure. MR has been widely described in the medical literature and has identified many significant causal relationships between multiple exposures and outcomes (Park et al., 2021b).

Even though IHD is also referred to as coronary artery disease (CAD) (Khan et al., 2020) and an MR study found little evidence to support a causal effect of AAM on the risk of CAD (Cao and Cui, 2020), women exhibit less physically obstructive CAD and relatively more preserved left ventricular function while having higher rates of myocardial ischemia and mortality than men with the corresponding age adjustment (Shaw et al., 2009; Smilowitz et al., 2011). Sex-specific pathophysiology of myocardial ischemia, including coronary microvascular dysfunction, a feature of the “Yentl Syndrome,” appears to be connected to this paradoxical sex difference. In light of this, the term IHD, as opposed to CAD, or coronary heart disease (CHD), is more appropriate for a topic that is special to women. Furthermore, even though Zheng et al. similarly recognized that women were more susceptible to the ischemia symptoms caused by myocardial infarction (MI) and discovered a genetic connection between earlier AAM and higher risk of MI by two-sample MR analysis (Zheng et al., 2022), studies of body mass index (BMI) and gender were neglected when evaluating the impacts of confounding or mediating factors. In our two-sample MR, we changed the outcome (CAD or MI) to the broader one (IHD) and used the external data source or multivariable MR analysis to further explore the effects of confounders, especially BMI and sex. Consequently, we explored the causal relationship between AAM and IHD *via* two-sample MR analysis to update the aforementioned studies, despite having a similar theoretical basis or starting point.

## Materials and method

### Study overview

**FIGURE 1:**
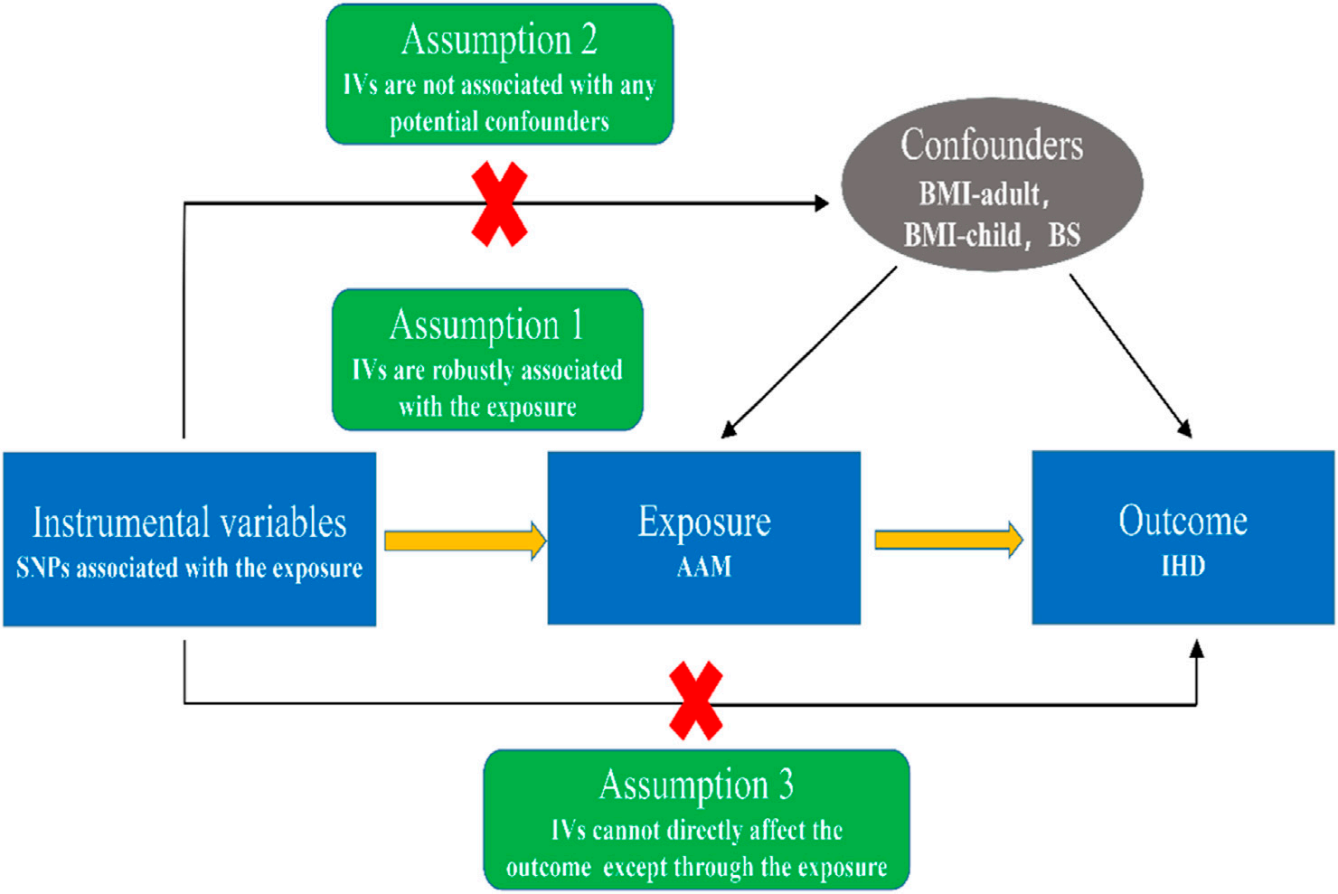
Schematic diagram showing the assumptions of Mendelian randomization analysis. Abbreviations: SNPs, single nucleotide polymorphisms; IVs, instrumental variables; AAM, age at menarche; IHD, ischemic heart disease; BMI, body mass index; BS, biological sex.

The fundamental study concept for the two-sample MR analysis is shown in Figure 1. In brief, when single nucleotide polymorphisms (SNPs) are used as instrumental variables (IVs) to probe the causal relationship between exposure (AAM) and outcome (IHD), there are three assumptions needed to be satisfied in this study: (1) IVs are strongly associated with age at menarche; (2) IVs should not be associated with confounders in the exposure-outcome association; (3) IVs should influence the outcome only *via* exposure instead of other pathways.

### Data source

The GWAS summary databases of exposure (AAM) and outcome (IHD) were obtained from the MR-base database (https://gwas.mrcieu.ac.uk/) (Hemani et al., 2018b). We filtered the MR-base database for the European population up to December 2021 using “menarche”, and “age when periods started” as keywords for the exposure database, and then did the same with “ischemic heart disease ", “cardiac ischemia” as keywords for the outcome database. If there are multiple GWAS databases, we prioritized GWASs with the maximum number of SNPs, largest sample sizes, and the year closer to now. (Supplementary Table S1; databases were accessed in January 2022).

### Selection of instrumental variables

The genetic IVs linked with exposure (AAM) were collected from the GWAS summary database with a sample size of 218,796 Europeans, and we underwent a variety of quality control procedures in our analysis to choose suitable instrumental SNPs that showed significant relationships with AAM. Firstly, we identified the SNPs based on the genome-wide significance threshold (*p* < 5 × 10^−8^), and then we removed highly correlated variants with $r^{2}>$ 0.001 to avoid linkage disequilibrium (LD) in the range of 10000 KB (Savage et al., 2018; Park et al., 2021a). All of these SNPs have minor allele frequencies (MAF) over 0.01, indicating a small statistical bias caused by poor confidence (Chen et al., 2022). Secondly, the F-statistic was calculated for each IV using the following formula to determine its strength: F = $R^{2}\frac{N-2}{{1-R}^{2}}$ (Burgess et al., 2011), where $R^{2}$ is the proportion of the variability of the AAM explained by each IV and N the sample size of the GWAS for the SNP-AAM association. F-statistic greater than 10 is recommended to avoid using the weakly genetic tool (Burgess and Thompson, 2012). To calculate $R^{2}$ for each IV, we used $R^{2}$ = 2 × EAF × (1 − EAF) × betaˆ2/(2 × EAF × (1 − EAF) × betaˆ2 + 2 × EAF × (1 − EAF) × se × N × betaˆ2) to obtain it (Shim et al., 2015; Papadimitriou et al., 2020). Among this formula, EAF, beta, se, and N represent the effect allele frequency, effect size, standard error, and sample size, respectively. Eventually, after extracting SNPs associated with exposure from the outcome, some SNPs not having related information in the outcome were eliminated. These quality control steps were completed to ensure the independence of the IVs and exclude the influence of linkage disequilibrium (LD) on the result (Hemani et al., 2018b).

### Statistics process

Four MR approaches were utilized as the primary statistical model, including fixed-effects inverse variance weighting (fe-IVW) (Burgess et al., 2013), multiplicative random-effects inverse variance weighting (mre-IVW) (Bowden et al., 2017), weighted median (WM) (Bowden et al., 2016b), and MR-Egger regression methods (Bowden et al., 2015), to evaluate the potential causal effect of AAM on IHD. Without taking into account the intercept term, IVW considered the weight as the reciprocal of the outcome variance (the square of standard error). Under the premise of IVW, we assume that IVs are not pleiotropic. Therefore, while utilizing the IVW approach, we must make sure that these IVs are not pleiotropic; otherwise, the findings were skewed (Bowden et al., 2015). Through its intercept test, the MR-Egger analysis may spot horizontal pleiotropy (Bowden et al., 2016a). Even if IVs are pleiotropic, the MR-Egger method can provide a more conservative appraisal of causal effects, and the resulting statistics are not susceptible to exaggeration (Burgess and Thompson, 2017). The WM method allowed for up to 50% of the variables in the SNPs to be non-valid instrumental variables, so it can consistently evaluate the causal effects (Bowden et al., 2016b).

To assess potential violations of the model assumptions in the MR analysis, we conducted a sensitivity analysis. First, we performed a heterogeneity test using Cochran’s Q statistic from IVW (along with the $I^{2}$ statistic), which may show that the heterogeneity is caused by pleiotropy or another factor (Greco et al., 2015). Cochran’s Q statistic roughly follows a x^2^ distribution with k-1 degrees of freedom (k is the number of genetic variations) (Liu et al., 2013). $I^{2}=\frac{\left(Q-\left(k-1\right)\right)}{Q}\times 100\%$ , the results can be divided into four intervals (0–25%, 25–50%, 50–75%, and 75–100%), and each interval corresponds to low, moderate, large and extreme heterogeneity (Liu et al., 2013). It would be difficult to directly combine IVs if there is heterogeneity (P_Q_<0.05). Second, since IVs do not follow MR’s presumptions when they may directly affect the outcome without exposure, the amount of pleiotropy in the test findings will cause significant bias in MR. (Hemani et al., 2018a; Ong and MacGregor, 2019), thus we employed MR pleiotropy residual sum and outlier (MR-PRESSO) to locate outliers and check the level of pleiotropy (Ong and MacGregor, 2019). To ensure adequate verification, we still used the MR-Egger intercept to test the pleiotropy. To further identify whether a single SNP drove causality, we used leave-one-out (LOO) analysis to reassess causal effects by sequentially excluding one SNP. The result we want indicates that the causal effect we found is dependable and robust because there is no discernible variation (Noyce et al., 2017). Power calculations were performed with the online tool mRnd (https://shiny.cnsgenomics.com/mRnd/) based on the outcome sample size, proportion of cases, $R^{2}$ sum, and a type I error rate of 0.05 (Freeman et al., 2013).

For the treatment of confounders, we took two different approaches. In the first one, after searching for pleiotropic SNPs of confounders in PhenoScanner V2 (Kamat et al., 2019), we used the remaining IVs for MR analysis after excluding certain IVs that were significantly associated (*p* < 5 × 10^−8^) with potential confounders (i.e., risk factors for IHD). In the second one, multivariable MR (MVMR) is carried out for certain important confounders (e.g. BMI). According to a recent paper with a similar starting point to ours (Zheng et al., 2022), smoking habits, blood pressure, lipids, blood glucose and so on, play mediating roles in the relationship between AAM and myocardial infarction (MI), but they did not specifically examine the mediation of BMI, and it has been observed that AAM causes adult obesity with childhood BMI adding to the pleiotropy (Gill et al., 2018), so we selected BMI included both adult body mass index (https://gwas.mrcieu.ac.uk/datasets/ebi-a-GCST006368/) and childhood body mass index (https://gwas.mrcieu.ac.uk/datasets/ebi-a-GCST90002409/) to conduct MVMR analysis for providing estimates separate from the effects of potential confounders. Additionally, though the GWAS data of the age at menarche is just finished in the females, other databases within our study contain both genders, we attempted to add biological sex (https://gwas.mrcieu.ac.uk/datasets/ebi-a-GCST90013474/) as a confounder in our MVMR analysis. The screening method for the GWAS summary databases of confounders was the same as for the database of exposure and outcome.

Our analyses are conducted by R software (version 4.1.3). Specially, we used the “TwoSampleMR” R package (version 0.5.6) and the “MRPRESSO” R package to perform MR analysis. The level of statistical significance was set at 0.05 in our study.

## Result

### Instrumental variables

After identifying at the genome-wide significance threshold (*p* < 5 × 10^−8^) and clumping (r^2^ ≤ 0.001), there are 125 SNPs remaining (Supplementary Table S2) with each SNP corresponding to an F-statistic >10 (range from 84.06 to 404.26). However, three SNPs (rs6548237, rs12607903, rs10063744) were not available in the summary data for IHD, and one SNP (rs56283944) extracted two kinds of information from the exposure data (Supplementary Table S3). After the harmonizing process, one SNP (rs56283944, β.IHD = 0.185, *P*.IHD = 0.218) was eliminated for incompatible alleles and 5 SNPs (rs1874984, rs4818008, rs61779780, rs6451675, rs9956387) were removed for being palindromic with intermediate allele frequencies. Finally, 117 SNPs were selected for subsequent analyses.

### Causal effect of age at menarche on ischemic heart disease

There is a negative correlation between AAM and the risk of IHD, increase in genetically predicted AAM was associated with a lower risk of genetically predicted IHD, with an odds ratio (OR) of 0.80 [95% confidence interval (CI) 0.72–0.88, *p* < 0.001] in the fixed IVW MR analysis, mre-IVW or WM MR analysis has similar result (OR = 0.80,95% CI:0.70–0.90, *p* < 0.001; OR = 0.79, 95% CI: 0.66–0.93, *p* = 0.006). Meanwhile, we have high power to identify the effect of AAM on IHD (100% power to identify an OR of 0.80, minimum and maximum detectable OR with 80% power: 0.9656/1.0348).

Although the MR-Egger regression did not show a significant correlation between AAM and the risk of IHD (*p* > 0.05), the power of the test of the MR-Egger regression analysis was considered to be relatively low compared to the other methods (Saade et al., 2011), so the above results suggested that later AAM is related with decreased risk of IHD (Supplementary Figures S1, S2; Supplementary Table S4). The funnel plot shows that when individual SNPs are used as IVs, the distribution of causality is roughly symmetrical (Supplementary Figure S3), suggesting that the results obtained by using 117 SNPs were sufficiently consistent and were not expected to be impacted by potential deviations.

Based on IVW, we conducted a heterogeneity analysis by Cochran’s Q statistic. The Q-statistic is 160.5(*p* = 0.004, $I^{2}=27.7\%$ ), indicating that there might be moderate heterogeneity. Then, to find and exclude the outliers, we chose to conduct the MR-PRESSO analysis. We found two outliers, rs10832013 (RSSobs = 0.002, *p* = 0.047) and rs9361178 (RSSobs = 0.003, *p* = 0.023), which might influence the causal effect of IVs. After their removal, the *p*-value increased from 0.004 to 0.089 for the MRPRESSO global test, which indicated that the pleiotropy was removed, and the P_Q_ was also increased to 0.090, suggesting that the heterogeneity has been removed. However, when we performed MR-Egger to further check the horizontal pleiotropy of our MR analysis, the result displayed that the intercept term was -0.006 (Supplementary Figure S4), which was not statistically significant with zero (*p* = 0.160 > 0.05), indicating that there was no horizontal pleiotropy between IVs. We used LOO analysis to examine if the causal effect changed in the presence or absence of the outlier, the LOO plots indicated that the causal estimation was robust in MR analysis (Supplementary Figure S5), and suggested that there was no single SNP that drove the causal relationship. Although there might be differences in the results of the above sensitivity tests, it generally suggested that individual SNP heterogeneity was largely balanced.

Using the online tool PhenoScanner V2, we found 50 SNPs from those 117 SNPs that could not be associated with any potential confounders (blood pressure, blood lipids, body mass index, etc.). Although there were no associations between the 50 SNPs and IHD (IVW, *p* = 0.09), when we selected and excluded one outlier (rs9361178) by performing MR-PRESSO analysis, the relationship between AAM and IHD re-appeared (IVW, OR = 0.80, 95% CI: 0.66—0.97, *p* = 0.022) (Supplementary Figure S6A), the intercept of MR-Egger analysis was not statistically significant with 0 (*p* = 0.129 > 0.05) (Supplementary Figure S6C), and the results from LOO method also showed that the association is robust (Supplementary Figure S6D). In the multivariable MR analysis, the relationship between AAM and IHD was attenuated after adjustment of BMI, including both adult and childhood BMI (OR = 0.86, 95% CI: 0.74 1.00, *p* = 0.045; OR = 0.83, 95% CI: 0.72 0.96, *p* = 0.01), even though it was remained significant after adjusting for biological sex (*p* < 0.001), indicating that sex differences barely affect the correlation between AAM with IHD and that BMI from childhood to adult mediates this association (Supplementary Table S5).

## Discussion

The MR analyses employed in this study provided strong evidence for the association between genetically predicted AAM and genetically predicted risk of IHD, but the MVMR analysis showed that this causation could be mediated by confounders, such as adult and child BMI.

Many observational studies, as we know, have been designed to explore the association between AAM and IHD. In the cohort research enrolling 867 White women with college degrees, Cooper et al. (1999) found that the risk of IHD reduced with increasing age of menarche onset (age-adjusted RR 0.76 per year, 95% CI: 0.6–0.95). Higher age at menarche was associated with a mean 6.0% (95% CI 1.2–10.6) decreased mortality from IHD (*p* = 0.01) in the 12-year cohort research conducted in the United States, although the authors did not display or discuss the findings after controlling for significant confounders like BMI (Jacobsen et al., 2009). According to a study through meta-analysis and seven observational studies, the pooled RR for IHD mortality was 0.969 (95% CI: 0.947–0.993) for every 1-year rise in AAM, with considerable heterogeneity (I^2^ = 44.9%, *P*_heterogeneity_ = 0.092), but in studies with excellent quality, lengthy follow-up (>12 years), and body mass index adjustment, heterogeneity appeared to decline (Chen et al., 2019).

In line with our results about confounders, some findings indicated that the associations between early menarche and cardiovascular health might be mainly driven by its associations with BMI (Elks et al., 2013; Bubach et al., 2018; Zheng et al., 2021). A study using both observational methods and MR analysis has concluded that AAM had an influence on obesity and cardiometabolic traits, and suggested that preventive interventions should instead focus on reducing childhood obesity (Bell et al., 2018). Additional MR study also found that for each 4 kg/m^2^ increase in BMI, observational estimates suggest a 26% increase in the odds of IHD, while causal estimates indicate a 52% increase (Nordestgaard et al., 2012). Therefore, further work is needed to detect the underlying mechanisms and the main targets of interventions.

There are several notable strengths of our MR analysis. Firstly, the outcome group is using the most recent broadly-defined IHD with a large sample size for the first time; Secondly, we used F-statistic to ensure that the IV used are strongly genetic tools, which was not previously used in a similar MR study (Cao and Cui, 2020). Thirdly, compared to the previous similar MR analysis (Cao and Cui, 2020; Zheng et al., 2022), we switched the outcome (CAD or MI) to the wider one (IHD), removed confounders by employing an external database (PhenoScanner V2) and utilized MVMR analysis to further investigate the mediating effects of BMI and sex. Finally, our work adds to the body of well-founded information supporting the need for greater investigation into the processes underpinning the early and late effects of AAM in IHD.

Also, our study has some limitations. Firstly, since the age at menarche estimations were provided voluntarily, they are subject to recall bias (Perry et al., 2014). Secondly, we were unable to obtain all sex-specific databases from the public datasets. Although the age at menarche is a sex-specific variable, we used summary-level genetic data from both sexes for IHD and confounders, assuming that the same genetic variations govern age at puberty in both men and women. Results from an LD score regression to determine the genome-wide genetic link between the age at menarche in girls and the age at voice broke in boys revealed a significant positive correlation, suggesting that comparable variations are responsible for controlling the timing of puberty in both sexes (Day et al., 2015a). We included biological sex (BS) as a confounder in our MVMR analysis, which showed a strong negative association between AAM and IHD even after adjusting for BS (*p* < 0.001) (Supplementary Table S4). Hence, the effect of sex is likely to be minimal and polygenic for the wide range of IHD, and further MR studies may yield more accurate and reliable results if gender-specific data are available to validate our results in women only. Thirdly, all participating participants were from Europe, so follow-up work is needed to explore whether the findings of this study can be generalized to other ethnic groups. For example, the index/proxy AAM SNPs at NUCKS1 and TMEM38B were most strongly associated in the Hispanic/Latina subsample (Fernandez-Rhodes et al., 2018), and there are significant inter-ethnic differences exist in allele frequencies of certain genes, which lead to differences in the age of menarche among different ethnic groups (Dvornyk and Waqar-ul-Haq, 2012). Finally, the MR approaches presupposed linearity of the modelled correlations, however, several observational investigations revealed a nonlinear relationship between AAM and cardiometabolic illnesses (Day et al., 2015b). Our MR estimations of the impact of AAM on the IHD may be seen as a causal effect that is representative of the entire population. To handle nonlinearity in MR, both parametric and nonparametric approaches have been proposed (Burgess et al., 2014; Silverwood et al., 2014).

## Conclusion

Our MR studies show an association between increased levels of genetically predicted AAM and a decreased risk of genetically predicted IHD, and this relationship was discovered to be mediated by both adult and childhood BMI. These findings may assist public health policymakers and physicians in developing more scalable and effective strategies to reduce the incidence of IHD due to early AAM without enacting political and social reforms, given the growing number of adolescent females worldwide who are presently threatened by earlier AAM as a result of social-economic progress. Additional interventional studies should be carried out to determine whether restricting BMI in females with a history of earlier AAM reduces their risk of IHD, and to gain a better understanding of other relevant mediators as well as how they interact.

## Data Availability

Publicly available datasets were analyzed in this study. This data can be found here: MR-base database (https://gwas.mrcieu.ac.uk/), age at menarche (https://gwas.mrcieu.ac.uk/datasets/ukb-a-315/), ischemic heart disease (https://gwas.mrcieu.ac.uk/datasets/finn-b-I9_IHD/), PhenoScanner V2 (http://www.phenoscanner.medschl.cam.ac.uk/), adult body mass index (https://gwas.mrcieu.ac.uk/datasets/ebi-a-GCST006368/), childhood body mass index (https://gwas.mrcieu.ac.uk/datasets/ebi-a-GCST90002409/), biological sex (https://gwas.mrcieu. ac.uk/datasets/ebi-a-GCST90013474/).
