# Supplementary material for: Age at menarche and ischemic heart disease: An update mendelian randomization study
Source: Front Genet. 2022 Nov 3;13:942861. doi: 10.3389/fgene.2022.942861 (PMC9671358; doi:10.3389/fgene.2022.942861)
Supplement: Supplementary file 1 [file DataSheet1.zip › supplementary material figures.docx]

**Age at Menarche and** **Ischemic Heart Disease: An update Mendelian randomization study**

Jing Chen^1^**^†^**, Heng Chen^2^**^†^**, Qiaozhen Zhu^3^, Qiannan Liu^1^, Yan Zhou^1^, Lan Li^4^*, Yan Wang^4^*


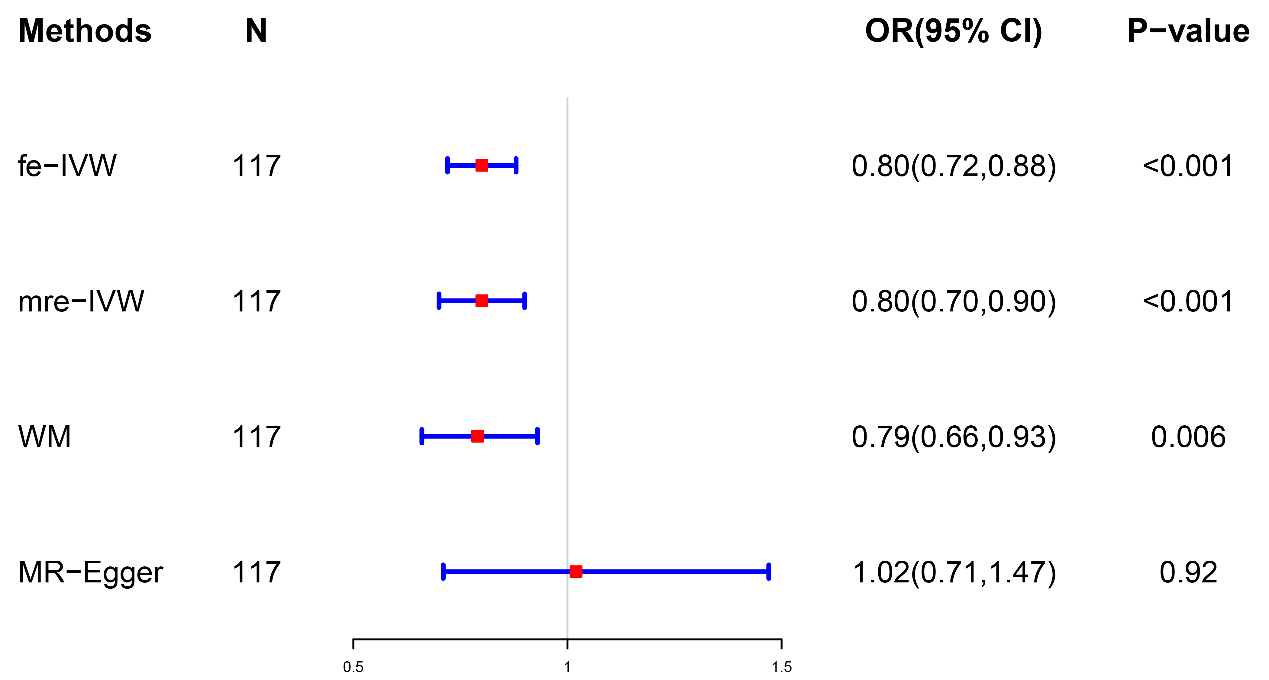


Figure 1 Forest plot of the two-sample MR analysis.

**Abbreviations**: CI, confidence interval; OR，odds ratio；N represents the number of single nucleotide polymorphisms used as instrumental variables; fe-IVW, fixed-inverse variance weighting; mre-IVM, multiplicative random-effects inverse variance weighting; WM, weighted median; MR, Mendelian Randomization.


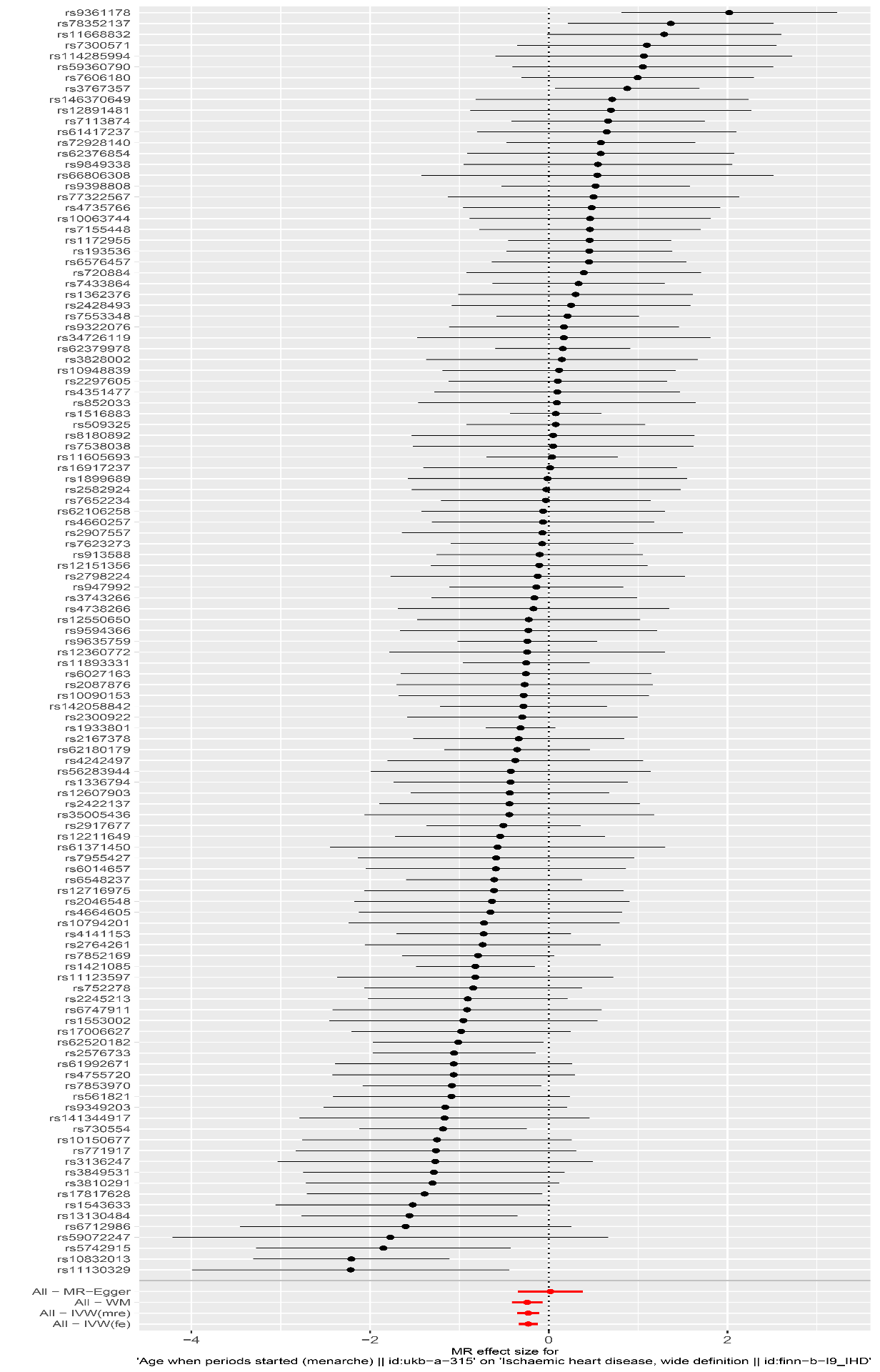


Figure 2 Associations between 117 SNPs and IHD

**Abbreviations**: IVW(fe), fixed-inverse variance weighting; IVM(mre), multiplicative random-effects inverse variance weighting; WM, weighted median; MR, Mendelian Randomization.


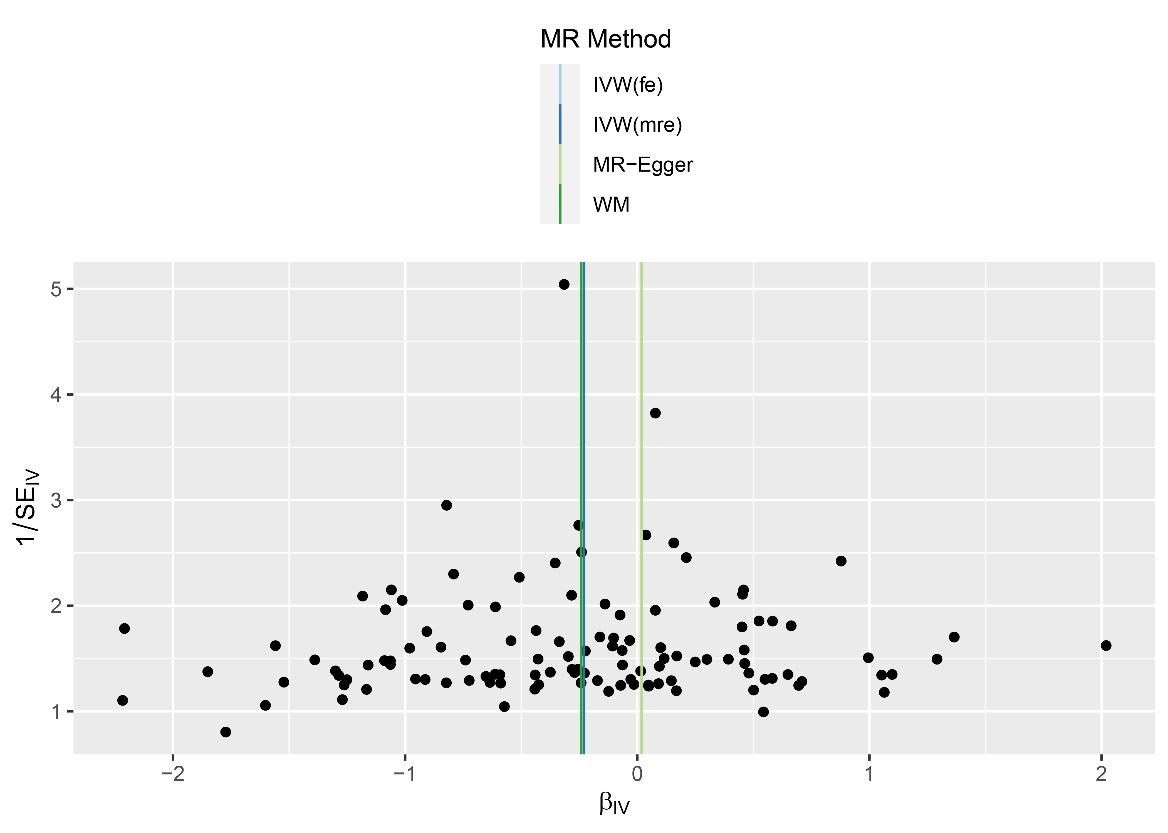


Figure 3 Funnel plot of the two-sample MR analysis.

**Abbreviations**: MR, Mendelian Randomization; SE, standard error; β, beta; IV, instrumental variables; IVW(fe), fixed-inverse variance weighting; IVM(mre), mre-IVM, multiplicative random-effects inverse variance weighting; WM, weighted median.


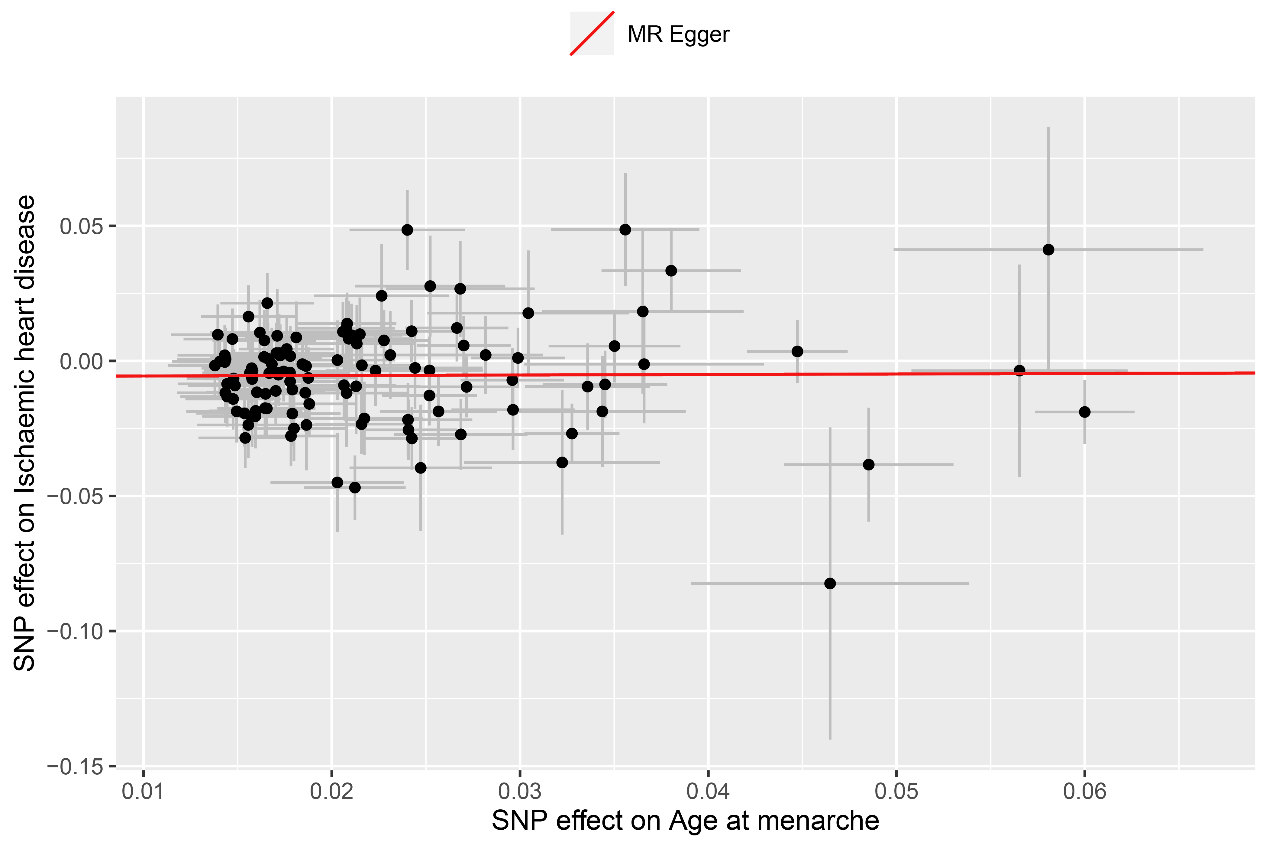


Figure 4 Scatter plot of MR-Egger regression.

**Abbreviations**: MR, Mendelian Randomization; SNP, single nucleotide polymorphism.


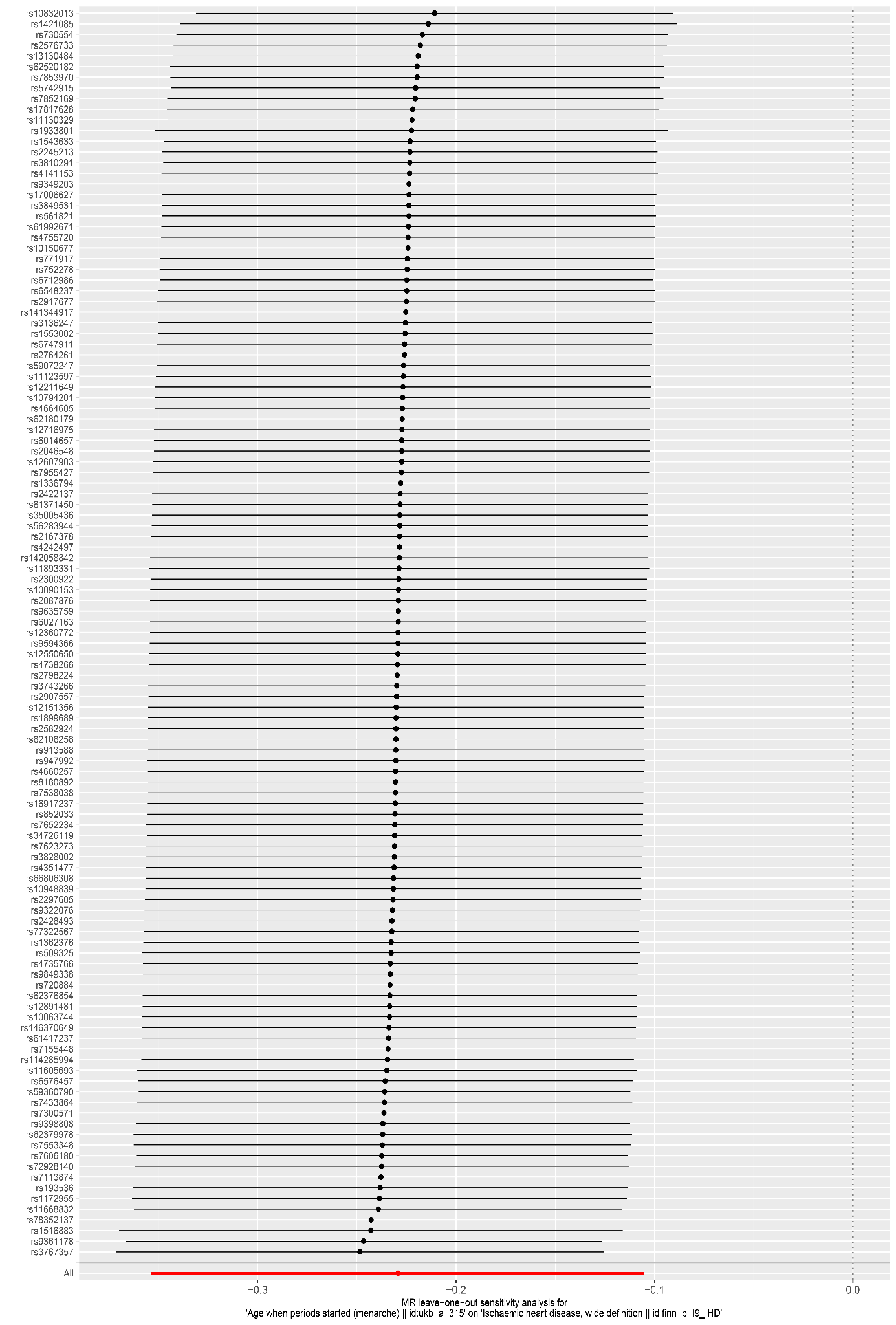


Figure 5  Result of “leave-one-out” sensitivity analysis
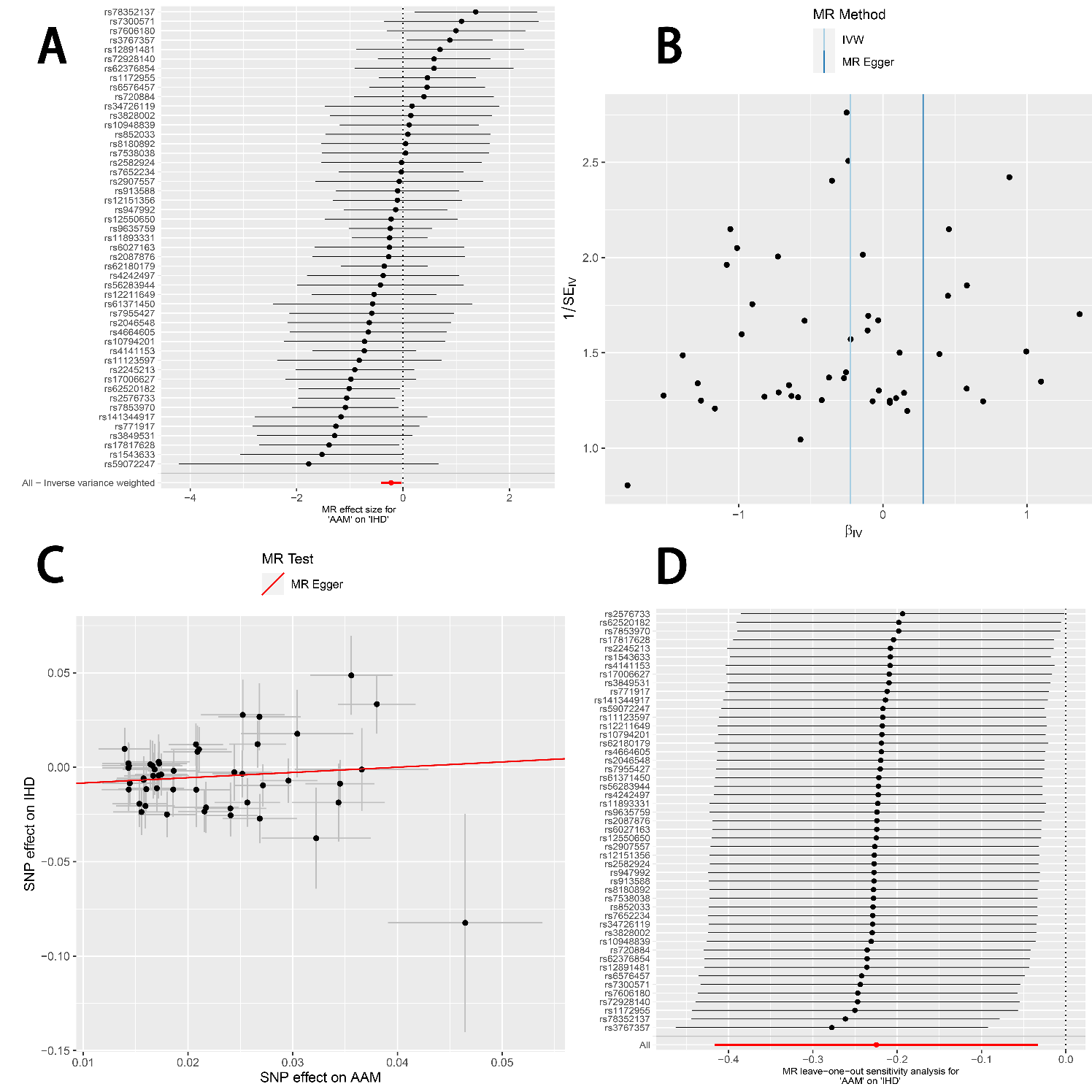


Figure 6 Summary of the MR analysis for 49 SNPs of AAM on IHD. (A) MR effect size of each IV and IVW. (B) The funnel plot of the causal effect of the 49 SNPs on IHD. (C) The scatter plot of causal effects of the 49 SNPs on IHD. (D) MR leave-one-sensitivity analysis for 49 SNPs on IHD.

**Abbreviations**: MR, Mendelian randomization; AAM, age at menarche; IHD, ischemic heart disease; IV, instrumental variable; IVW, inverse variance weighting; SNP, single nucleotide polymorphism.
